# COVID-19 workplace adaptation in Ireland: the development and validation of a quantitative survey

**DOI:** 10.1136/bmjph-2024-001825

**Published:** 2026-02-26

**Authors:** Yanbing Chen, Carolyn Ingram, Penpatra Sripaiboonkij, Elizabeth Alvarez, Carla Perrotta, Conor Buggy

**Affiliations:** 1 College of Aviation, Embry-Riddle Aeronautical University, Daytona Beach, Florida, USA; 2 School of Public Health, Physiotherapy and Sports Science, University College Dublin, Dublin, Ireland; 3 School of Public Health, Physiotherapy and Sports Science; Centre for Safety and Health at Work, University College Dublin, Dublin, Ireland; 4 Department of Health Research Methods, Evidence and Impact (HEI), McMaster University, Hamilton, Ontario, Canada

**Keywords:** COVID-19, Mental Health, Public Health, Epidemics, methods

## Abstract

**Introduction:**

The purpose of this study was to develop and validate a new survey instrument to evaluate employee adaptation during the COVID-19 pandemic in Ireland, with the intention that the instrument could be adapted for use in future pandemic scenarios.

**Methods:**

The survey was developed iteratively by a multidisciplinary research team based on five previously identified key themes: (1) support received from the organisation, (2) adaptation pressure, (3) work–life balance, (4) health condition and (5) workload/working hours. From November 2021, employees from six organisations participated in a two-round pilot study (total n=589). Exploratory factor analysis (EFA) was conducted using data from the first round in IBM SPSS V.27 to refine the survey items, followed by confirmatory factor analysis (CFA) in IBM AMOS V.24 using data from the second round to test the factor structure.

**Results:**

The initial survey demonstrated good internal consistency (n=63, Cronbach’s α=0.963). EFA identified a five-dimension structure corresponding to the predefined themes. After the removal of 19 items, the initial model fit was suboptimal (χ²/df=2.17, p<0.001; Comparative Fit Index (CFI)=0.864; standardised root mean square residual (SRMR)=0.066; root mean square error of approximation (RMSEA)=0.079). Subsequent CFA-based refinements improved the model fit substantially. The final model consisted of 25 items across five dimensions, with acceptable fit indices (χ²/df=1.76, p<0.001; CFI=0.926; SRMR=0.061; RMSEA=0.064).

**Conclusions:**

This study presents the first validated survey instrument specifically designed to evaluate employee adaptation experiences during the COVID-19 pandemic. The survey provides a valuable tool for human resources and occupational health professionals to monitor employee well-being during periods of organisational and environmental disruption. Furthermore, the selected items can be adapted for broader application across different working settings and future public health emergencies.

**WHAT IS ALREADY KNOWN ON THIS TOPIC**

- The COVID-19 pandemic forced widespread workplace adaptations that significantly affected employee health and well-being, yet existing measurement tools focused narrowly on remote work, stress or self-efficacy rather than overall employee adaptation.

**WHAT THIS STUDY ADDS**

- This study develops and validates a comprehensive, standardised survey instrument that captures multiple dimensions of employee adaptation to pandemic-related workplace disruption, informed by the distinct COVID-19 experience in Ireland.

**HOW THIS STUDY MIGHT AFFECT RESEARCH, PRACTICE OR POLICY**

- This study provides an adaptable tool that enables researchers to examine employee adaptation across diverse workplaces, supports occupational health and human resource professionals in designing targeted postpandemic interventions and offers policymakers evidence to incorporate workplace perspectives into future public health emergency planning.

## Introduction

The onset of the COVID-19 pandemic had the potential to impact all industries and occupations globally. To maintain business continuity while ensuring employee safety, various new measures were introduced to workplaces worldwide. Surveillance measures (eg, symptomatic or asymptomatic testing), outbreak investigations and response (eg, contact tracing and quarantine), environmental adjustments (eg, minimising social interactions and employees’ physical proximity), education initiatives (eg, training on infection prevention and control measures), changes in work arrangements (eg, transition to remote work) and the widespread use of face masks and other mandatory personal protective equipment (PPE).^1^ Workplace adaptation is defined as ‘the act or process of changing to better suit a situation’.

Early research into the health and safety implications from the pandemic indicates that the changes brought about in work arrangements had impacted employees’ health and well-being depending on the type of arrangement—work-from-home (WFH) or essential workers (who conduct a range of operations and services in industries that are essential to ensure the continuity of critical functions), the mitigation strategies used and the way they were communicated and implemented.^1^

Among WFH employees, adverse psychological outcomes (eg, isolation, loneliness and anxiety) were observed when adapting to the new COVID-19 related measures.^2 3^ For example, a compulsory WFH policy reduced face-to-face interactions with colleagues and increased the workload of essential workers.^4^ The WFH arrangement may contribute to mental health issues caused by feelings of isolation and reduced socialisation.^5^ WFH may have a negative impact on overall work-life balance, thereby decreasing job satisfaction.^6^ However, some employees fully embraced WFH, as they could quickly adapt to a more flexible way of working.^7^ The transition from workplaces to remote working at home required employees to create a new working space and to adapt to different modes of communication as well as new working processes—the pandemic forced this level of flexibility that was often untested. This ‘forced flexibility’, if coupled with a lack of support from the organisation or ineffective communication caused by WFH, could become a challenge for workers leading to negative effects on their well-being.^4^

For those who continued working in essential services on the frontline of pandemic response (healthcare, emergency services and those industries deemed essential by governments), the heightened perception of the COVID-19 contagion risk was associated with emotional exhaustion,^8^ the degree of which varied depending on workplace demographics.^1^ In addition, essential workers worried about transmitting the virus to their family members at home after their working day (Brooks *et al*,^9^ 2020). Apart from the impact new workplace adaptations had on employees’ physical and mental health, workplace disturbance and uncertainty over business continuity as socioeconomic impacts were felt as the pandemic unfolded also led to career-related fears among workers.^10^ Given the constant changes within workplaces throughout a pandemic, it is necessary for occupational safety and health (OSH) professionals to understand the impact workplace disruption has had on employees.

In Ireland, the first reported case of COVID-19 occurred on 29 February 2020.^11^ At the outset of the pandemic, Ireland focused on isolation and social distancing to limit as much as possible the spread of infection. The series of lockdowns was extensive and gave Ireland a distinct experience during the COVID-19 pandemic which can provide significant lessons as we continue to analyse and reflect on the positive steps taken along with the challenges that can be used to tackle future pandemics.^12^ The country entered the initial lockdown to ‘flatten the curve’ of infection cases from 27 March to 31 May 2020. There were subsequent lockdowns in autumn/winter 2020 and the first half of 2021. These lockdowns resulted in a significant reduction in the spread of the virus.^13^ However, adverse effects have started to show from these measures, which included isolation, including an increase in mental health issues.^9 14^ Like many European countries, COVID-19 guidance in Ireland was typically issued following the release of similar policies from the WHO. Among the control measures taken in place, PPE usage, especially the usage of surgical masks in hospital wards, is considered a cornerstone of effective infection control practice. As most decisions on health emergencies in the country are largely made by public health experts with limited consideration from a workplace perspective, policies are primarily focused on reducing transmission, protecting vulnerable populations and maintaining the healthcare workforce.^15^ For example, testing and contact tracing were essential measures in managing infections in Ireland, but COVID-19 testing criteria for different populations were amended during the pandemic, leading to periods of confusion.^16^ As reported, occupational health and human resource (HR) professionals were not confident about preparations made in their organisations to cope with the emergence of COVID-19 in Ireland at the very beginning, but this cohort soon became key personnel who were crucial to the development of successful emergency planning measures.^15^

The instruments used to evaluate employee adaptation during COVID-19 primarily focus on remote-work capability and self-efficacy, such as digital self-efficacy measures^17^ and the E-Work Life Scale.^18^ Other tools developed during the pandemic, including the COVID-19 Stress Scales,^19^ assess stress and anxiety caused by the pandemic. However, there was no standardised measurement instrument for the overall evaluation of employee adaptation during COVID-19 or indeed one that can be used in other pandemic scenarios. Therefore, this study has developed such an instrument involving participants in Ireland which has the potential to be useful for other cultural contexts in case of similar public health emergencies in the future.

## Methods

**Figure 1:**
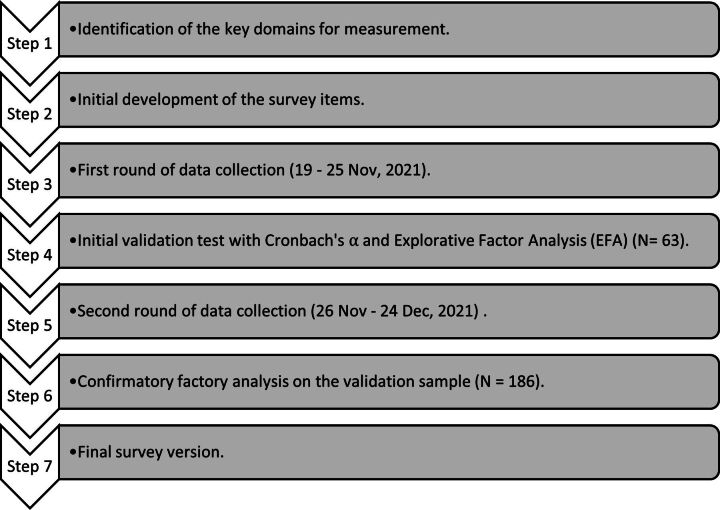
Schematic of the seven-step survey development and validation workflow.

Our larger research project was funded by Science Foundation Ireland (now Research Ireland) as part of their COVID-19 Rapid Research Project funding. According to research protocol, our established project has included a systematic review on COVID-19 workplace control measures, a survey developed to explore workplace COVID-19 control measures in different cultural contexts and a qualitative study phase using focus group interviews with OSH and/or HR professionals from Irish occupational settings to further investigate employee COVID-19 adaptation difficulties. During the research process, we found that a tool to measure and monitor behavioural apathy, occupational fatigue and adherence to protective measures is needed in the workplace. Thus, this study aims to develop and validate such a survey instrument, following the implementation of seven main research steps, illustrated in figure 1.

### Initial survey development

Effective emergency response at the workplace involves planning for the hierarchy of control measures including engineering controls, administrative controls and the use of PPE.^20^ Among those, engineering controls aim to separate employees from a hazard and associated risk(s), such as the separation of symptomatic employees from others to halt the spread of the disease or the separation of staff from customers using plastic or glass barriers.^21^ Administrative controls limit the number of employees in working areas through social distancing,^22^ for example, implementing teleworking strategies, reducing working hours or even temporarily shutting down the workplace.^23^ PPE (eg, face masks) can effectively prevent the spread of infection in settings where people work in close contact with others.^24^ Periodic surveillance, either symptomatically or with testing, if tests are available, also demonstrated effectiveness in protecting employees even in the context of high community transmission.^1^ Beyond the implementation of preventive and protective measures, effective emergency planning requires the establishment of policies to mitigate financial and other pandemic risks (eg, paid sick leave, childcare planning) and ongoing communication of and workforce involvement in an organisation’s evolving emergency response.^22^

Based on a review of the existing literature that included the earliest research on the workplace adaptation to the pandemic,^1^ we have developed a multilingual survey to map the COVID-19 prevention and control measures used in global workplaces.^25^ Subsequently, a suite of 15 focus groups was conducted with OSH professionals and/or HR professionals from a diverse range of occupational sectors and organisation sizes (n=60).^26^ The participants recruited were categorised into groups by work sectors and organisation size classified by the Irish Central Statistics Office (eg, small=10–49 employees; medium=50–249 employees and large=250+ employees). Building on these findings,^1^ employees’ difficulties during COVID-19 adaptation in this study were investigated from four perspectives: adaptation challenges, protections, availability of support from the organisation and communication efficiency.

A preliminary questionnaire with 40 items was developed to measure workers’ occupational experiences including fatigue and attitudes towards pandemic communication and implemented control measures in their workplace. The questions were further redefined in an iterative process after three rounds of critical discussions between multidisciplinary researchers (eg, OSH, medicine, psychology and public health), ensuring the content reflected the aforementioned four themes that emerged from the focus groups with occupational health management on disruptions and adaptations during the COVID-19 pandemic.^15^ A Likert scale (0–10) was chosen as the question/answer structure for the reason of user friendliness and future usability.

**Figure 2:**
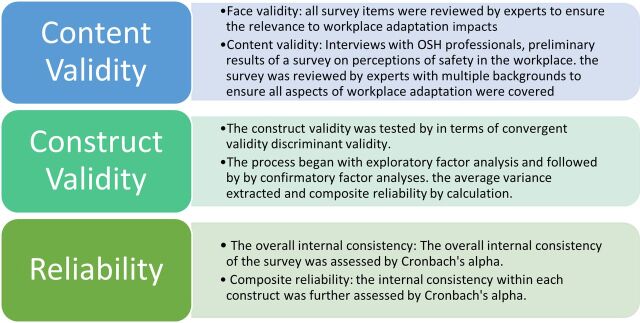
Validation and reliability. OSH, occupational safety and health.

The questionnaire instrument proposed for the validation stage covered all themes in 40 items under four sections and could be completed between 10 and 15 min. Among the questions developed, 32 of the questions were phrased in a positive way and 8 items were phrased in a reversed (negated) way with the aim of reducing response bias.^22^ Figure 2 presents the efforts made to ensure the validity and reliability of the survey in multiple steps.

### Participants

**Figure 3:**
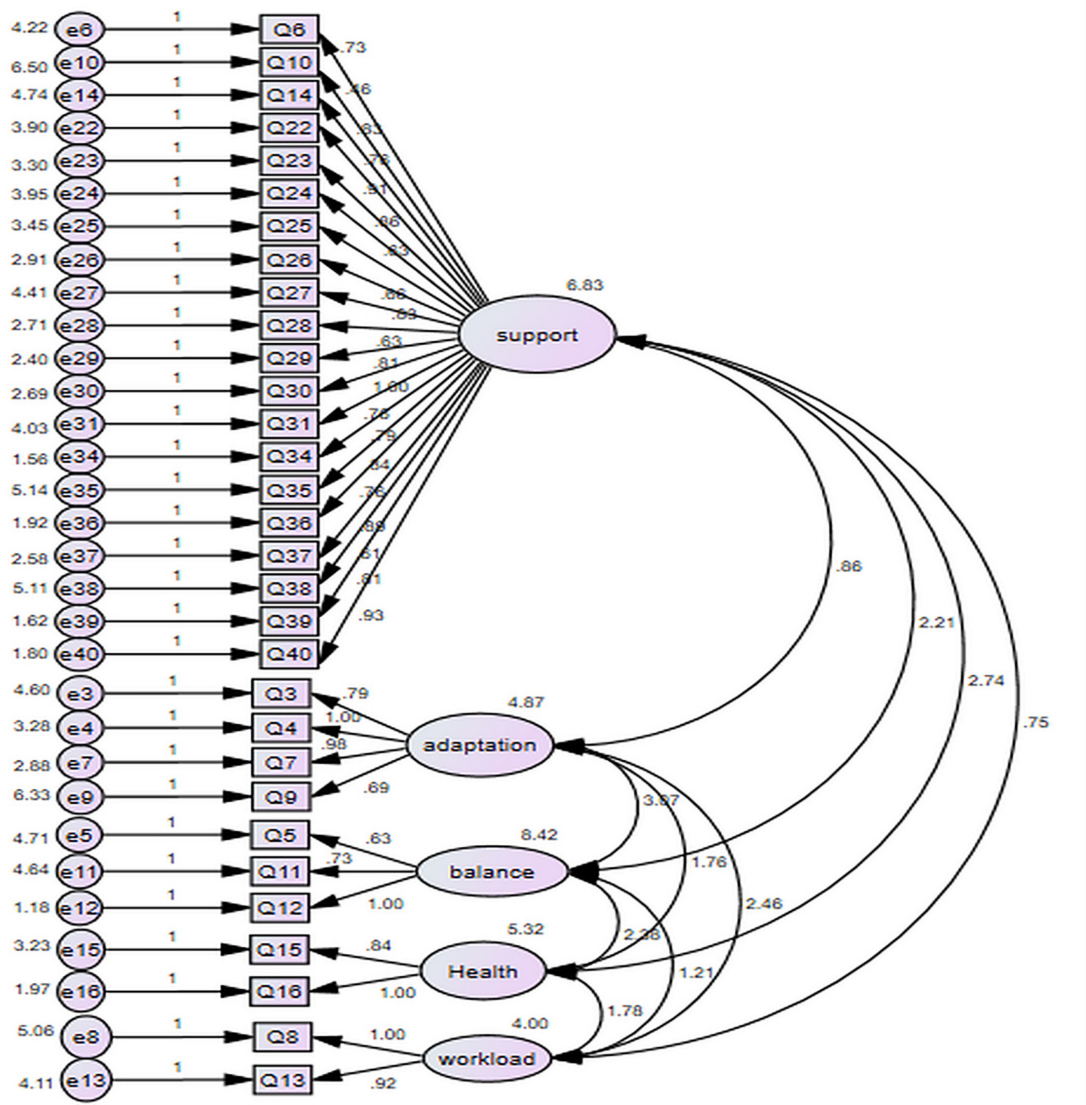
Initial CFA model (31 items before adjustment). CFA, confirmatory factor analysis.

Six organisations agreed to take part in the validation stage. In total, 589 workers (63.1% male, 69.1% age 41∼50 and 55% working length >11 years) responded in two rounds for validation testing from six participating organisations in Ireland across public (transportation, local authority and education) and private sectors (construction and consulting). In case the initial survey developed may require further adjustment, the first round of data was preliminarily analysed after 2 weeks of survey dissemination. However, no major changes were made to the survey according to the preliminary survey feedback. It was thus proceeded to the second round of data collection for another 2-week long period, with a reminder email sent by the OSH professionals in each participating organisation. Therefore, the participants involved in the first round of data collection (19–25 November 2021) were not involved in the second round of data collection (26 November to 24 December 2021), so there was no duplicated data between the two samples. Cases with any missing value among the 40 survey items to be validated were excluded for analysis. The demographic information for participants who completed the 40 survey items in each of the two samples is in table 1, and the response rate in each sector is presented in figure 3.

**Table 1:** Sample characteristics

Demographic variables	First round data collection (N=63)		Second round data collection (N=186)	
	n	%	n	%
Gender				
Male	34	54.0	123	66.8
Female	29	46.0	58	31.5
Prefer not to say	0	0.0	3	1.6
Age				
18–30	8	12.7	22	11.8
31–40	16	25.4	40	21.5
41–50	19	30.2	67	36.0
51–60	16	25.4	45	24.2
60+	4	6.3	12	6.5
How many years have you worked in this organisation?				
0–5	31	50.0	58	31.2
6–10	4	6.5	18	9.7
11+	27	43.5	110	59.1
Sectors				
Private	19	30.2	44	23.7
Public	44	69.8	142	76.3

### Data analysis

All the data downloaded from Qualtrics^XM^ were entered into IBM SPSS (V.27) for analysis. After data cleaning, all responses of negatively formulated items were reversed (online supplemental table 1). Given that the survey framework had not been used in previous quantitative research, it is recommended to begin with an exploratory factor analysis (EFA) to explore the underlying factor structure of the measure, followed by a confirmatory factor analysis (CFA) to further validate the structure identified.^27^

10.1136/bmjph-2024-001825.supp1 Supplementary data

#### First round data analysis

After the first round of data was collected between 19 November 2021 and 25 November 2021, reliability analysis was performed (n=63) in SPSS, using Cronbach’s alpha with the threshold of 0.70.^28^ Subsequently, EFA was undertaken to identify data structures that met the criteria for simple structure and to confirm that item loadings were theoretically coherent. There are no a priori assumptions about relationships among the items included in the psychometric tool when using EFA. The Kaiser-Meyer-Olkin Measure of Sampling Adequacy (KMO) is a statistic that indicates the proportion of variance in variables that might be caused by underlying factors.^29^ High values (>0.60) generally indicate that a factor analysis may be useful with the data.^30^ Bartlett’s test of sphericity tests the hypothesis that the correlation matrix is an identity matrix, which would indicate that the variables are unrelated and therefore unsuitable for structure detection.^31^ P values less than 0.05 indicate that a factor analysis may be useful with the data.^32 33^ Initial selection of the survey dimensions was based on eigenvalues >1.^34^

#### Second round data analysis

To test the framework identified from Round One, further analyses were performed on the Second Round of data collected from 26 November to 24 December 2021, using CFA analysis. CFA is a statistical technique allowing the researcher to test the hypothesis that a relationship between observed variables and their underlying latent constructs exists. Specifically, the survey items that successfully passed through the initial validation were included in a CFA model to test both the quality of the overall structural model and the item composition of the measurement scales. For model estimation, the maximum likelihood method was applied using Amos V.24. This stage also aimed to iteratively refine the model using the information provided by modification indices to improve model fit. This includes statistical indicators such as the ratio of model χ^2^ to the df (χ^2^/df), Comparative Fit Index (CFI), root mean square error of approximation (RMSEA) and standardised root mean square residual (SRMR).

According to these indices introduced by research literature, the χ^2^ value is a first ‘absolute index’ for evaluating the model fit to the data and assesses the magnitude of discrepancy between the sample and fitted covariance matrices.^35^ In this study, the χ^2^/df was adopted since this corrected form of χ^2^ can minimise the impact of sample size sensitivity on the index. The CFI is a ‘relative’ fit index which compares the χ^2^ for the hypothesised model to one from a ‘baseline model’, in which all of the variables are uncorrelated.^35^ CFI higher than 0.80 is considered acceptable and higher than 0.95 is considered very good. SRMR and RMSEA indices are two ‘absolute indices’ providing information of the statistical error about the model in terms of the data entered.^35^ Previous studies show that SRMR is more sensitive to misspecifications in covariances,^36^ while RMSEA is more sensitive to model specification, df and sample size.^37^ Values of both statistics smaller than 0.08 are usually considered acceptable.

In a CFA, convergent and discriminant validity examine the extent to which measures of a latent variable shared their variance and how they are different from others.^38^ The convergent validity of the measurement model can be assessed by the average variance extracted (AVE) and composite reliability (CR) by calculation in Microsoft Excel. AVE measures the level of variance captured by a construct versus the level due to measurement error. Values above 0.7 are considered very good, whereas the level of 0.5 is acceptable. CR is a less biased estimate of reliability than Cronbach’s alpha; the acceptable value of CR is 0.6 or above. The AVE for construct can be calculated as follows:

$$AVE=\frac{\sum_{i=1}^{k}\lambda i^{2}}{\sum_{i=1}^{k}\lambda i^{2}+\sum_{i=1}^{k}Var\left(e_{i}\right)}$$

The CR formula^39^ is as follows:

$$CR=\frac{{\left(\sum_{i=1}^{k}\lambda_{i}\right)}^{2}}{{\left(\sum_{i=1}^{k}\lambda_{i}\right)}^{2}+\sum_{i=1}^{k}Var\left(e_{i}\right)}$$

Among the two formulas above, k is the number of items, $\lambda_{i}$ is the factor loading of item i and $Var\left(e_{i}\right)$ is the error variance of the k^th^ indicator (k = 1, …, k) of construct.

$$\sum {\;}_{i=1}^{k}Var\left(e_{i}\right)=\sum {\;}_{i=1}^{k}1-\lambda_{i}^{2}$$

The value of the square root of the AVE for each construct should be greater than the correlation involving the constructs to ensure discriminant validity.^38^

## Results

### Initial survey validation

#### Internal reliability test

The results indicated the initial 40 items survey had very good internal consistency (n=63, Cronbach’s alpha=0.963). As shown in online supplemental table 2, it was not necessary to delete any items as the overall reliability was very high and would not have been improved by item deletion.

#### Exploratory factor analysis on the survey prototype

The initial EFA using the first round of responses (n=63) indicates that all 40 items were suitable for dimension deduction (KMO=0.784, p<0.001), with 76.6% of total variance explained. Specifically, analysis was conducted using principal components analysis in SPSS with varimax rotation. Thereafter, factor selection was based on interpretation of the scree plot in which components with an eigenvalue larger than 1 were retained. Items that doubled loaded >0.4 or did not load on any factor >0.4 were removed.

As indicated in online supplemental table 3, Q1, Q2, Q17, Q18, Q20, Q32 and Q33 were deleted since they were double loaded >0.4. Q19 and Q21 were also removed as they did not have sufficient loading in any of the dimensions. Thus, the validity of the survey was improved by deleting the nine items, and the remaining 31 questions were divided into five dimensions:

- Dimension 1: support received from the organisation (Q6, Q10, Q14, Q22–31, Q34–40).
- Dimension 2: adaptation pressure (Q3, Q4, Q7, Q9).
- Dimension 3: work-life balance (Q5, Q11, Q12).
- Dimension 4: health condition (Q15, Q16).
- Dimension 5: workload/working hours (Q8, Q13).

#### Calibration of survey domains with CFA (second round of sample)

After the second round of data collection, CFA was performed on the 31 remaining items (n=186) using SPSS Amos. An independent cluster model containing the five dimensions with the 31 items was tested using a confirmatory approach with the second sample of responses. According to the criteria of the indices, the model fit was not satisfied (figure 2): χ^2^/df = χ^2^ (919)/df (424) = 2.17, p<0.001, CFI=0.864, SRMR=0.066, RMSEA=0.079.

**Figure 4:**
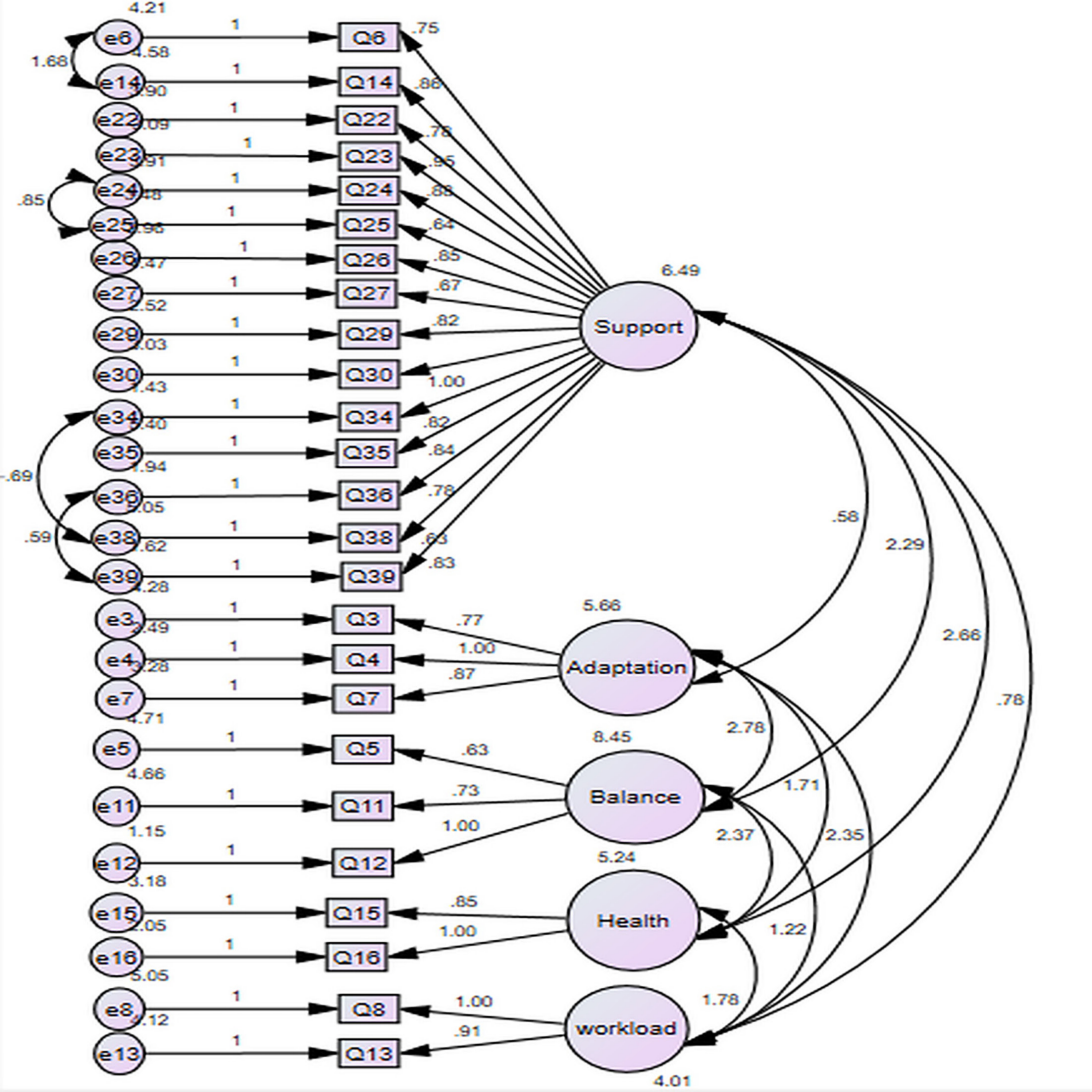
Final model after adjustment according to modification indices.

Thereafter, a post hoc model fitting was conducted on inspection of modification indices (with standardised residuals if necessary) to refine the framework. For example, the largest modification index (MI) was obtained between e27 and e28 (MI=33). Considering other MIs in relation to these two factors, Q28 was the appropriate choice to be removed to improve the overall model fit. Items were also deleted if their loadings were less than 0.5 to meet convergent validity.^38^ Based on such indices and after assessment of item content, Q28 was thus removed. By repeating such procedures, 10 amendments were made in total, including deletion of six items (Q9, Q10, Q28, Q31, Q37 and Q40) and adding covariance for four pairs of parameters (e6<–>e14, e24<–>e25, e34<–>e38 and e36<–>e39). Finally, a model presenting acceptable statistical fit indices was achieved (figure 4): χ^2^/df = χ^2^ (459)/df (261) = 1.76, p<0.001, CFI=0.926, SRMR=0.061, RMSEA=0.064 (online supplemental table 4).

In figure 3, generated with Amos V.24, rectangles are observed variables and ovals are latent traits. The latent trait is considered to predict its observed variables, hence the direction of the arrows. Each observed variable in the measurement model has an error term associated with it, such as e6, e14. The error terms impact the variables they point to. A curved double-headed arrow signifies a covariance between two variables. The absence of arrows is also a specific prediction hypothesising no correlation between the unlinked variables.

#### Analysis of measurement model

The acceptability of the measurement model was assessed by AVE, CR, internal consistency within each construct and discriminant validity. Those indices are reported in online supplemental table 5 for the five constructs of the remaining 25 items following the model fit adjustment. In general, AVE greater than 0.5, CR and Cronbach’s alpha greater than 0.7 indicate good reliability of the model.^28^ Except for the ‘Workload’ construct, all other four constructs met recommended criteria. As indicated, the model constructs reported reciprocal correlations with Pearson’s r coefficient ranging from a maximum of 0.437 (p<0.01; between ‘Support’ and ‘Workload’) to a minimum of 0.086 (not significant; between ‘Adaptation’ and ‘Support’). As the square root of the AVE value of each construct was greater than the correlation between that construct and other ones, the model indicates good discriminant validity.

## Discussion

In this study, a Likert 0–10 scale survey measuring COVID-19 workplace adaptation impacts was developed and refined. For example, it is easy to observe important statistical indicators such as means and medians. Also, the 11-point scale survey can be easily tweaked into other formats (eg, 7-point or 5-point scale) to meet OSH professionals’ specific need for their workplace. Among the 40 items in the initial designed questionnaire, 9 items were deleted during EFA and 6 items were deleted during the CFAs. The final 25-item framework resulted in an acceptable model fit and can be used as a conceptual framework and measurement tool to evaluate the impacts of COVID-19 workplace adaptations on employees. Overall, the framework underpinning the adjusted survey has a good model fit (figure 4) which supports the further use of the survey in occupational settings.

Dimension 1 describes ‘support from the organisation’ measured by 15 items (Q6, Q14, Q22-27, Q29, Q30, Q34-36, Q38 and Q39). Among those, the loading of Q30 ranks highest, indicating that whether workers felt comfortable seeking support from their organisation if they were concerned had the most significant impact on evaluating employees’ satisfaction on organisational supports in relation to the pandemic. Positive covariance exists between four pairs of variables in this dimension, which means both variables in each pair (Q6 and Q14, Q24 and Q25, Q34 and Q38, and Q36 and Q39) have the same tendency when being evaluated. For example, participants tend to give similar ratings on Q6 ‘I was given the supports that I needed to adjust to COVID-19 measures at work’ and Q14 ‘My organisation provided sufficient supports to facilitate home working when appropriate during the pandemic’.

In Dimension 2 ‘adaptation pressure’ (Q3, Q4 and Q7), Q4 has the highest loading, indicating that the extent to which employees feel exhausted when adapting work to follow COVID-19 measures can significantly reflect the degree of pressure experienced during COVID-19 adaptation. However, as the covariance value between Dimension 1 and Dimension 2 is not high, it can be interpreted that current available supports from organisations are unlikely to alleviate employees’ adaptation pressure during the pandemic. This is also confirmed in online supplemental table 5 showing that the correlation between the two constructs is not significant, aligning with previous studies reporting that WFH employees have limited opportunities to receive support from their organisation.^4^

Q12 was the most influential indicator in Dimension 3 which illustrates work-life balance (Q5, Q11 and Q12). Specifically, employees consider it is important that adapting to new working arrangements creates a better work-life balance. Dimension 4 evaluates employee health from both physical and mental aspects (Q15 and Q16), which is quite straightforward.

Dimension 5, regarding workload/working hours (Q8 and Q13), has the overall lowest loading (0.445) as a construct. We chose not to eliminate this dimension at this stage because workload or working hours have always been a key indicator for employee well-being.^40^ The result may relate to the fact that employees had difficulty calculating their daily hours as they had to layer household chores in between. However, this topic may require further discussion as COVID-19 has provided an unexpected circumstance in which employees have an opportunity to adjust their working hours.^41^

The survey built on existing qualitative research that formed the initial stages of this research project which was conducted with OSH professionals from a wide variety of workplace settings in Ireland during the COVID-19 pandemic, and enabled researchers to understand COVID-19 adaptation impacts in workplaces from the perspective of OSH professionals and their colleagues/fellow employees. The questionnaire instrument presented in this paper, developed by a group of experts from multiple disciplines, was tested and adjusted using a series of recognised analysis methods, supported OSH professionals to customise the intervention programmes to their respective organisation for the purpose of improving employee well-being after COVID-19 workplace adaptation.

A purely quantitative survey is efficient for measuring broad trends, especially when employees are in the middle of coping with the pandemic, but it lacks the depth needed for understanding why employees respond the way they do. From our experience in the development of this instrument as part of the larger research project, a mixed-methods approach offers richer insights by incorporating qualitative data that contextualises numerical findings, helping institutions develop more targeted, effective strategies for supporting employee adaptation during COVID-19. Thus, we recommend that an interview or potentially focus groups should be followed if the respondent(s) agrees to participate, as qualitative data can provide more context and nuanced answers to help organisations develop response strategies in addition to the survey results.^42^

A quantitative method requires sufficient response rate, which could be difficult as employees may be overwhelmed by changes caused by the pandemic. One approach is to have trusted figures, such as OSH and/or HR professionals, initiate the survey and interviews, as employees are more likely to participate when communication comes from familiar and credible sources. Additionally, using multiple formats (eg, online surveys, brief interviews or mobile-friendly options) and offering flexible participation times can help reach workers across different roles and schedules. Providing clear explanations of how the results will be used, maintaining confidentiality and sharing follow-up actions can further build trust and encourage participation.

Timing is critical when surveying employees during a pandemic, as collecting feedback too early or too late can limit the usefulness of the data. Ideally, a survey should be administered 2–6 weeks after a major change in work arrangements in response to a public health emergency. This window allows employees enough time to experience the new conditions and recognise what has fundamentally changed, while still being able to clearly articulate the challenges they face and the support they need from the organisation. Immediately after a shift, employees may still be in the process of adapting and unable to reflect meaningfully on their needs. Conversely, waiting too long can lead to a situation where employees have unknowingly adjusted to unhealthy routines or work patterns, potentially masking early signs of poor adaptation. In such cases, issues like stress, burnout or other mental health concerns may already be emerging.^43^ Surveying within this optimal timeframe ensures that organisations capture accurate, actionable insights to support employee well-being and guide timely interventions.

At the early stages of the pandemic, most research has focused on how healthcare sectors, such as nursing homes or hospital departments,^44^ prepared for COVID-19 prevention or treatment. Up until this research was initiated, few studies have focused on general working environments, such as building sites, food-processing plants and high-volume office, transportation or retail settings that proved to be at increased risk for COVID-19 outbreaks.^45^ This survey can be used in these industries, and the data collected can provide organisations with valuable insights that translate directly into practical applications for supporting employees during pandemics. These insights can guide organisations in shaping internal interventions, such as improving ergonomic support, enhancing mental health resources or adjusting workloads. For example, results may highlight the need for clearer WFH workload guidelines to prevent employees from burnout when working remotely. In most countries, policies are mainly developed by public health experts with a focus on population-level health protection. However, as an example, when classifying the COVID-19 virus in the context of the Biological Agents Directive,^46^ a panel of public health experts downplayed the aerosol transmission route and the contributing factors related to contagiousness in working conditions.^47^ The employee-level insights from this survey may not be fully used unless OSH professionals are actively involved in policy discussions.^15^

As a limitation, although the participants in this study were from multiple workplaces in both public and private sectors, a total of 6 organisations participated in the survey validation test and the data collected may not be sufficient to represent all the industries in Ireland even though a significant effort was made to recruit as many organisations as possible from the 60 that took part in the qualitative focus groups. Furthermore, limited to the access to the target population and research time frame, essential workers and those who WFH were not distinguished in this survey. Thus, the survey did not capture the variation across different working arrangements. In data analysis, to avoid bias caused by missing data, observations with incomplete responses were removed before analysis, so using only complete cases may also introduce bias. Given that the enterprise size of the participating organisations varies, it was not applicable to compare differences across organisations. Meanwhile, factors such as specific work arrangement, age or gender may influence how employees adapt and we recognise that additional studies using this developed questionnaire are required to further test the validity, reliability and generalisability of the instrument, especially in other cultural contexts (eg, Asian countries with totally different systems and approaches of management regarding public health emergencies at workplaces).

## Conclusion

This is the first study aiming to quantitatively evaluate adaptation impacts on employees caused by the COVID-19 pandemic in Ireland. Future research can focus on other countries and other experiences in public health emergencies, such as MERS-CoV and H1N1. More research is recommended on how organisations can better support employee adaptation caused by public health emergencies, especially for those who are sent home to work. Also, more studies are required to explore a new working model which enables employees to have more flexibility about working style resulting from the COVID-19 pandemic. Furthermore, the long-term impacts among employees post COVID-19 should be investigated using a longitudinal design based on the framework developed in this study. Additionally, the validation of the survey can be further tested with larger populations within Ireland as well as in other countries and regions. The questionnaire (31 items) in its current form (online supplemental appendix 2) will be made available for public usage at the conclusion of the project, and all our coauthors agree that other researchers will then be free to use the survey, to adapt or refine the instrument to customise its use to different contexts. In conclusion, the survey developed in this study has the potential to improve employee well-being during the pandemic by customised intervention, with a potential to be applied in a wider range of working settings internationally.

## Data Availability

All data relevant to the study are included in the article or uploaded as supplementary information.
