# Supplementary material for: COVID-19 workplace adaptation in Ireland: the development and validation of a quantitative survey
Source: BMJ Public Health. 2026 Feb 26;4(1):e001825. doi: 10.1136/bmjph-2024-001825 (PMC12993357; doi:10.1136/bmjph-2024-001825)
Supplement: Supplementary data [file bmjph-4-1-s001.pdf]

Appendix 1 Supplemental Tables

Table 1 Initial Survey items designed across the four dimensions

Themes and items	Positively formulated items	Negatively formulated items
**Theme 1 – Adaptation**	1, 2, 5, 6, 11, 12, 15, 16	3, 4, 7, 8, 9, 10, 13
**Theme 2 – Protection**	17, 18, 19, 20, 22, 23, 24, 32, 33	21
**Theme 3 – Availability of Support**	14, 25, 26, 27, 28, 29, 30	/
**Theme 4 – Communication Efficiency**	31, 34, 35, 36, 37, 38, 39, 40	/

Table 2 Internal reliability of the 40 survey items developed

Items (Cronbach's Alpha = 0.963, N=63)	Corrected Item-Total Correlation	Cronbach's Alpha if Item Deleted
1. My working environment improved during the pandemic	.647	.962
2. COVID-19 safety measures made my work easier	.613	.962
3. I found the workplace changes brought about by the pandemic challenging	.544	.962
4. I felt exhausted when adapting my work to follow COVID-19 measures	.560	.962
5. I felt that the pandemic allowed me time to evaluate my work-life balance	.458	.963
6. I was given the supports that I needed to adjust to COVID-19 measures at work	.816	.961
7. It was stressful adapting to COVID-19 safety measures at work	.404	.963
8. My working hours became longer due to the pandemic	.222	.964
9. COVID-19 safety measures made my work harder	.575	.962
10. I feel that safety culture has declined in my organisation compared to before the pandemic	.618	.962
11. I want the workplace changes brought about by the pandemic to stay	.318	.963
12. Adapting to new working arrangements introduced during the pandemic has given me a better work-life balance	.394	.963
13. My workload increased due to COVID-19	.203	.964
14. My organisation provided sufficient supports to facilitate home working when appropriate during the pandemic	.672	.962
15. I am satisfied with my physical health at present	.392	.963
16. I am satisfied with my mental health at present	.428	.963
17. COVID-19 safety measures at my workplace are clear and easy to understand	.789	.961
18. I feel fully aware of the COVID-19 risks that exist in my workplace	.583	.962
19. I was able to comply with COVID-19 safety measures when doing my job during the pandemic	.421	.963
20. The PPE I use at work to prevent the spread of COVID-19 makes me feel safer	.626	.962
21. I think the PPE to prevent the spread of COVID-19 caused other risks	.168	.964
22. I feel protected from COVID-19 at work	.698	.961
23. I believe that my safety is the priority of my organisation	.742	.961

Items (Cronbach's Alpha = 0.963, N=63)	Corrected Item-Total Correlation	Cronbach's Alpha if Item Deleted
24. My organisation considered my personal circumstances (family, disability, etc.) when prioritising safety during the pandemic	.763	.961
25. My organisation provided sufficient supports for those who contracted COVID-19	.691	.961
26. As the pandemic unfolded, my workplace responded quickly to the changing situation	.767	.961
27. I am clear on who is responsible for health and safety in my organisation	.660	.962
28. I always know where to find information about COVID-19 in my workplace	.789	.961
29. I have a clear understanding of my organisation's reasons for responding to the pandemic in the way that it did	.751	.961
30. I feel comfortable seeking support from my organisation when or if I have concerns about working conditions related to the pandemic	.772	.961
31. I was consulted in a timely fashion about my opinions on COVID-19 safety measures	.860	.960
32. I feel that as a team we are doing our best to keep each other safe	.678	.962
33. I feel that my colleagues are doing all they can to follow COVID-19 safety measures	.623	.962
34. My workplace provides good communication in relation to COVID-19	.812	.961
35. I have been fully briefed on responses to prior outbreaks in my workplace	.690	.961
36. COVID-19 safety measures are communicated in a way that is easy to understand	.793	.961
37. I feel that any feedback I provided on COVID-19 safety measures was valued by my organisation	.857	.960
38. My health and safety personnel often provide useful talks on COVID-19	.638	.962
39. COVID-19 safety measures were communicated in a timely fashion in my organisation	.810	.961
40. I trust my organisation to keep me as safe as possible from COVID-19 in my workplace	.788	.961

Table 3 The initial Exploratory Factor Analysis result based on the 40 items (19-25 Nov 2021)

Items (N=63, KMO=0.784, p<0.001, with 76.6% total variance explained)	Factor loadings in each dimension								Decision on the item
	1	2	3	4	5	6	7	8	
28. I always know where to find information about COVID-19 in my workplace	.876								Retained in Dimension 1
34. My workplace provides good communication in relation to COVID-19	.872								Retained in Dimension 1
39. COVID-19 safety measures were communicated in a timely fashion in my organisation	.849								Retained in Dimension 1
37. I feel that any feedback I provided on COVID-19 safety measures was valued by my organisation	.843								Retained in Dimension 1
29. I have a clear understanding of my organisation's reasons for responding to the pandemic in the way that it did	.814								Retained in Dimension 1
40. I trust my organisation to keep me as safe as possible from COVID-19 in my workplace	.804								Retained in Dimension 1
35. I have been fully briefed on responses to prior outbreaks in my workplace	.787								Retained in Dimension 1
27. I am clear on who is responsible for health and safety in my organisation	.781								Retained in Dimension 1
31. I was consulted in a timely fashion about my opinions on COVID-19 safety measures	.777								Retained in Dimension 1
30. I feel comfortable seeking support from my organisation when or if I have concerns about working conditions related to the pandemic	.745								Retained in Dimension 1
38. My health and safety personnel often provide useful talks on COVID-19	.732								Retained in Dimension 1
24. My organisation considered my personal circumstances (family, disability, etc.) when prioritising safety during the pandemic	.724								Retained in Dimension 1
23. I believe that my safety is the priority of my organisation	.720								Retained in Dimension 1

Items (N=63, KMO=0.784, p<0.001,	Factor loadings in each dimension								Decision on the
36. COVID-19 safety measures are communicated in a way that is easy to understand	.691								Retained in Dimension 1
6. I was given the supports that I needed to adjust to COVID-19 measures at work	.690								Retained in Dimension 1
26. As the pandemic unfolded, my workplace responded quickly to the changing situation	.688								Retained in Dimension 1
25. My organisation provided sufficient supports for those who contracted COVID-19	.670								Retained in Dimension 1
10. I feel that safety culture has declined in my organisation compared to before the pandemic	.636								Retained in Dimension 1
14. My organisation provided sufficient supports to facilitate home working when appropriate during the pandemic	.542								Retained in Dimension 1
17. COVID-19 safety measures at my workplace are clear and easy to understand	.504					.503			Discarded for cross loading
22. I feel protected from COVID-19 at work	.454								Retained in Dimension 1
3. I found the workplace changes brought about by the pandemic challenging		.858							Retained in Dimension 2
7. It was stressful adapting to COVID-19 safety measures at work		.851							Retained in Dimension 2
4. I felt exhausted when adapting my work to follow COVID-19 measures		.755							Retained in Dimension 2
9. COVID-19 safety measures made my work harder		.727							Retained in Dimension 2
2. COVID-19 safety measures made my work easier		.528	.449						Discarded for cross loading
1. My working environment improved during the pandemic	.496	.521							Discarded for cross loading
11. I want the workplace changes brought about by the pandemic to stay			.845						Retained in Dimension 3
12. Adapting to new working arrangements introduced during the pandemic has given me a better work-life balance			.819						Retained in Dimension 3

BMJ Publishing Group Limited (BMJ) disclaims all liability and responsibility arising from any reliance placed on this supplemental material which has been supplied by the author(s)

**Items (N=63, KMO=0.784, p<0.001,**	**Factor loadings in each dimension**								**Decision on the**
5. I felt that the pandemic allowed me time to evaluate my work-life balance			.712						Retained in Dimension 3
15. I am satisfied with my physical health at present				.899					Retained in Dimension 4
16. I am satisfied with my mental health at present				.844					Retained in Dimension 4
20. The PPE I use at work to prevent the spread of COVID-19 makes me feel safer				.483	.422			.439	Discarded for cross loading
33. I feel that my colleagues are doing all they can to follow COVID-19 safety measures	.511				.739				Discarded for cross loading
32. I feel that as a team we are doing our best to keep each other safe	.579				.668				Discarded for cross loading
19. I was able to comply with COVID-19 safety measures when doing my job during the pandemic						.758			Discarded as it is the only item in its dimension
18. I feel fully aware of the COVID-19 risks that exist in my workplace	.414					.494			Discarded for cross loading
8. My working hours became longer due to the pandemic							.851		Retained in Dimension 7
13. My workload increased due to COVID-19							.823		Retained in Dimension 7
21. I think the PPE to prevent the spread of COVID-19 caused other risks								.863	Discarded as it is the only item in its dimension

Notes: Varimax rotation converged in 7 iterations and loadings < 0.4 were suppressed

Table 4 The Common Structural Equation Modelling fit indices (Kline, 2005).

Fit indices	Description	Acceptable value ranges
Model chi-square (χ^2)	Assess overall fit and the discrepancy between the sample and fitted covariance matrices.	The lower the better, and values closer to 0 indicate a better fit.
X^2/df	Chi-square difference with 1 degree of freedom	Acceptable values range from 1 to 5
Comparative fit index (CFI)	A revised form of NFI. Not very sensitive to sample size. Compares the fit of a target model to the fit of an independent or null model	CFI ≥ 0.80 indicates an acceptable model fit
Root mean square error of approximation (RMSEA)	A parsimony-adjusted index. Values closer to 0 represent a good fit.	RMSEA < 0.08 indicates an acceptable model fit
Standardized Root Mean Square Residual (SRMR)	The square-root of the difference between the residuals of the sample covariance matrix and the hypothesized model. If items vary in range (i.e., some items are 1-5, others 1-7) then RMR is hard to interpret, better to use SRMR	SRMR <0.08

Table 5 Correlational and reliability statistics

Construct	AVE[1]	CR[2]	Cronbach's Alpha	Adaptation[3]	Balance[3]	Health[3]	Support[3]	Workload[3]
**Adaptation**	.566	.795	.788	**.753**				
**Balance**	.595	.811	.804	.086	**-.772**			
**Health**	.631	.773	.769	.337*	.284**	**-.794**		
**Support**	.561	.948	.948	.244	.411**	.276**	**-.749**	
**Workload**	.445	.616	.614	.344**	.437*	.211**	.340**	**-.667**

Notes: The bold numbers listed diagonally are the square root of the variance. The off-diagonal elements are the correlations among the constructs. For discriminate validity, the diagonal elements should be larger than the off-diagonal elements.
[1] AVE stands for Average Variance Extract.
[2] CR stands for Composite Reliability.
[3] Survey Dimensions: Adaptation Pressure; Work-life Balance; Health Condition; Support from the organisation; Workload/Working hours
* p<.05, ** p<.001, Pearson's r correlation calculated (2-tailed) by using SPSS.

Appendix 2 Questionnaire

Workers and COVID-19 – Evaluation of their experience

The purpose of this survey is to get your view on COVID-19 related safety measures taken in your organisation, whether you worked from home, at the workplace or in a hybrid/blended mode. Your answers are anonymous and will be used to assist the Occupational Safety and Health management to protect workers from COVID-19 and/or potential future respiratory infectious diseases. You can choose N/A option if you think the question is not applicable to your work.

Your gender? ☐ **Male** ☐ **Female** ☐ **Other: ___________** ☐ **Prefer not to say**

Your Age? ☐ **Under 18** ☐ **18-30** ☐ **31-40** ☐ **41-50** ☐ **51-60** ☐ **60+**

How many years have you worked in this organisation? ☐ **0-5** ☐ **6-10** ☐ **11+**

On a scale of 0 to 10 (with 0 being strongly disagree and 10 strongly agree) to what extent do you agree with the following statements.

	Strongly disagree				Neither agree nor disagree					Strongly Agree	
	**0**	**1**	**2**	**3**	**4**	**5**	**6**	**7**	**8**	**9**	**10**	**N/A**

1. I found the workplace changes brought about by the pandemic challenging
2. I felt exhausted when adapting my work to follow COVID-19 measures
3. I felt that the pandemic allowed me time to evaluate my work-life balance
4. I was given the supports that I needed to adjust to COVID-19 measures at work
5. It was stressful adapting to COVID-19 safety measures at work
6. My working hours became longer due to the pandemic

7. COVID-19 safety measures made my work harder
8. During the pandemic I feel that the safety culture declined in my organisation versus before
9. I want the workplace changes brought about by the pandemic to stay
10. Adapting to new working arrangements introduced during the pandemic has given me a better work-life balance
11. My workload increased due to COVID-19
12. My organisation provided sufficient supports to facilitate home working when appropriate during the pandemic
13. I am satisfied with my physical health at present

14. I am satisfied with my mental health at present
15. I feel protected from COVID-19 at work
16. I believe that my safety is the priority of my organisation
17. My organisation considered my personal circumstances (family, disabilities etc) when prioritising safety during the pandemic
18. My organisation provided sufficient supports for those who contracted COVID-19
19. As the pandemic unfolded, my workplace responded quickly to the changing situation
20. I am clear on who is responsible for health and safety in my organisation

21. I always know where to find information about COVID-19 in my workplace

22. I have a clear understanding of my organisation's reasons for responding to the pandemic in the way that it did

23. I feel comfortable seeking support from my organisation when or if I have concerns about working conditions related to the pandemic

24. I was consulted in a timely fashion about my opinions on COVID-19 safety measures

25. My workplace provides good communication in relation to COVID-19

26. I have been fully briefed on responses to prior outbreaks in

my workplace

27. COVID-19 safety measures are communicated in a way that is easy to understand
28. I feel that any feedback I provided on COVID-19 safety measures was valued by my organisation
29. My health and safety personnel often provide useful talks on COVID-19
30. COVID-19 safety measures were communicated in a timely fashion in my organisation
31. I trust my organisation to keep me as safe as possible from COVID-19 in my workplace
